# Quantitative study: should vacuum-assisted biopsy be the first biopsy approach in breast interventional techniques in stereotactic guided microcalcifications rather than 14 gauge core needle biopsy?

**DOI:** 10.1186/bcr3791

**Published:** 2015-11-05

**Authors:** Anuma Shrestha, Louise Wilkinson, Rosalind Given-Wilson, Judi Curtis

**Affiliations:** 1 St George's Healthcare NHS Trust, London, UK

## Introduction

Stereotactic guided 14 gauge core needle biopsy (14GCNB) and vacuum-assisted biopsy (VAB) are the two commonly used biopsy methods for obtaining an accurate diagnosis for microcalcifications. Retrospective review of 399 patients who underwent biopsy for breast microcalcification during screening assessment from April 2012 to March 2013 was used to evaluate the performance and cost-effectiveness of both methods.

## Methods

The repeat biopsy rate, diagnostic accuracy, time taken and cost of both methods was calculated. Microsoft Excel (2010) and SPSS 22 were used for statistical analysis.

## Results

The repeat biopsy rate for 14GCNB was 10 % and VAB was 6 %. Specificity, PPV and NPV were all 90 % or higher when compared against post-surgical final diagnosis in both methods. The sensitivity of VAB was 93.75 % vs. 71.88 % for 14GCNB for first biopsy. There was no significant difference in procedure time between two methods (*p *= 0.291). VAB necessitated almost double the rate of clip deployment compared with 14GCNB. The cost of VAB would be £69,922 greater than 14GCNB if used as the first-line biopsy method in this series.

## Conclusion

This study found VAB to have higher sensitivity than 14GCNB. There was also a trend for lower repeat biopsy rate, higher diagnostic accuracy and lower surgical upgrade with VAB. If VAB had been used as the first biopsy method for microcalcifications, the cost would have been significantly higher. 14GCNB is a cost-effective but less sensitive first biopsy method for selected microcalcifications.

